# Elucidating the Molecular Pathways and Therapeutic Interventions of Gaseous Mediators in the Context of Fibrosis

**DOI:** 10.3390/antiox13050515

**Published:** 2024-04-25

**Authors:** Aohan Li, Siyuan Wu, Qian Li, Qianqian Wang, Yingqing Chen

**Affiliations:** 1 Chronic Disease Research Center, Medical College, Dalian University, Dalian 116622, China; liaohan@s.dlu.edu.cn (A.L.); wusiyuan@s.dlu.edu.cn (S.W.); liqian@s.dlu.edu.cn (Q.L.); 2 Engineering Technology Research Center for The Utilization of Functional Components of Organic Natural Products, Dalian University, Dalian 116622, China

**Keywords:** gaseous mediators, fibrosis, oxidative stress, inflammation, myofibroblasts

## Abstract

Fibrosis, a pathological alteration of the repair response, involves continuous organ damage, scar formation, and eventual functional failure in various chronic inflammatory disorders. Unfortunately, clinical practice offers limited treatment strategies, leading to high mortality rates in chronic diseases. As part of investigations into gaseous mediators, or gasotransmitters, including nitric oxide (NO), carbon monoxide (CO), and hydrogen sulfide (H_2_S), numerous studies have confirmed their beneficial roles in attenuating fibrosis. Their therapeutic mechanisms, which involve inhibiting oxidative stress, inflammation, apoptosis, and proliferation, have been increasingly elucidated. Additionally, novel gasotransmitters like hydrogen (H_2_) and sulfur dioxide (SO_2_) have emerged as promising options for fibrosis treatment. In this review, we primarily demonstrate and summarize the protective and therapeutic effects of gaseous mediators in the process of fibrosis, with a focus on elucidating the underlying molecular mechanisms involved in combating fibrosis.

## 1. Introduction

Fibrosis represents the ultimate and widespread pathological consequence of numerous chronic inflammatory disorders, encompassing a diverse spectrum of medical conditions within the field of medicine. It serves as a common endpoint characterized by the excessive deposition of extracellular matrix components (ECM), culminating in tissue remodeling and functional impairment [1]. In fibrotic diseases, organs undergo a complex interplay between injury and subsequent repair processes. This intricate sequence of events involves various cellular and molecular mechanisms contributing to the development and progression of fibrosis in affected tissues. These processes entail the activation and recruitment of inflammatory cells, proliferation and activation of fibroblasts, deposition of extracellular matrix components, and remodeling of tissue architecture [2,3]. In the progression of fibrosis, a series of complex cellular events occur, contributing to the pathological remodeling of affected tissues. Fibroblasts become activated and undergo proliferation, subsequently differentiating into myofibroblasts. Additionally, epithelial cells initiate the process of epithelial–mesenchymal transition (EMT), transforming into fibroblasts and myofibroblasts. These cellular transitions are orchestrated by a multitude of factors, including the activation of the TGF-β signaling pathway, oxidative stress, inflammation, and cellular senescence, all of which play pivotal roles in driving the activation, proliferation, and ECM production by fibroblasts. This intricate interplay of cellular and molecular processes underscores the multifactorial nature of fibrosis and highlights the need for targeted therapeutic strategies to intervene at various stages of its progression [4,5,6]. Fibrosis-related diseases account for a substantial proportion of mortality associated with various medical conditions. However, the therapeutic options currently available for treating fibrotic diseases are limited, highlighting the critical need for the development of more effective strategies to address this significant healthcare challenge [3].

In the progression of fibrosis, a series of complex cellular events occur, contributing to the pathological remodeling of affected tissues. Fibroblasts become activated and undergo proliferation, subsequently differentiating into myofibroblasts. Additionally, epithelial cells initiate the process of EMT, transforming into fibroblasts and myofibroblasts. These cellular transitions are orchestrated by a multitude of factors, including the activation of the TGF-β signaling pathway, oxidative stress, inflammation, and cellular senescence, all of which play pivotal roles in driving the activation, proliferation, and ECM production by fibroblasts. This intricate interplay of cellular and molecular processes underscores the multifactorial nature of fibrosis and highlights the need for targeted therapeutic strategies to intervene at various stages of its progression [4,5,6]. Fibrosis-related diseases account for a substantial proportion of mortality associated with various medical conditions. However, the therapeutic options currently available for treating fibrotic diseases are limited, highlighting the critical need for the development of more effective strategies to address this significant healthcare challenge [3].

Recent research has elucidated the involvement of five bioactive gasotransmitters, namely nitric oxide (NO), carbon monoxide (CO), hydrogen sulfide (H_2_S), hydrogen (H_2_), and sulfur dioxide (SO_2_), in regulating oxidative stress, inflammation, apoptosis, and proliferation processes in various fibrotic conditions such as myocardial fibrosis, pulmonary fibrosis, liver fibrosis, and renal fibrosis. Both preclinical animal studies and clinical investigations have underscored the significant therapeutic potential of low-dose exogenous gas inhalation and intraperitoneal administration of gaseous donors, including compounds such as S-nitroso-N-acetyl-D,L-penicillamine (SNAP), sodium nitroprusside (SNP), carbon monoxide-releasing molecules (CORM), sodium hydrosulfide (NaHS), and hydrogen-rich water, in alleviating the progression of fibrosis [7,8,9,10]. These interventions exert their effects by modulating the TGF-β signaling pathway, oxidative stress, inflammation, cellular senescence, and apoptosis.

In this comprehensive review, we thoroughly evaluated and summarized the therapeutic impact of gaseous mediators on diverse fibrotic diseases, shedding light on their underlying molecular mechanisms involved in fibrosis development and progression, with particular emphasis on the roles of hydrogen (H_2_) and sulfur dioxide (SO_2_).

## 2. NO Either Suppresses or Promotes the Process of Fibrosis

Nitric oxide (NO) is a colorless, odorless, and tasteless gas that exists as a free radical at room temperature. It exhibits high reactivity and quickly reacts with oxygen in the atmosphere to form nitrogen dioxide. Within biological systems, nitric oxide plays a crucial role in signaling pathways and regulating functions such as vascular tone, neurotransmission, and immune responses [11]. Endogenous NO is generated by three isoforms of nitric oxide synthases (NOSs): neuronal NOS (nNOS; NOS1), inducible NOS (iNOS; NOS2), and endothelial NOS (eNOS; NOS3) [11]. Nitric oxide synthases (NOSs) catalyze the conversion of L-arginine into nitric oxide (NO) and citrulline [12]. Among these isoforms, neuronal NOS (nNOS) and inducible NOS (iNOS) are mainly located in the cytosol, while endothelial NOS (eNOS) is associated with the cellular membrane through palmitoylation and myristoylation processes [13]. NO possesses various biological functions, such as smooth muscle relaxation, blood pressure reduction, inhibition of vascular smooth muscle cell proliferation, suppression of platelet aggregation, and enhancement of the non-specific immune response [14]. Recent research has increasingly demonstrated that endogenous and exogenous NO, as well as its concentration, exhibit differential effects on various fibrotic diseases through distinct fibrotic mechanisms.

### 2.1. NO Regulates ROS Production and HSCs’ Apoptosis in Liver Fibrosis

**Figure 1:**
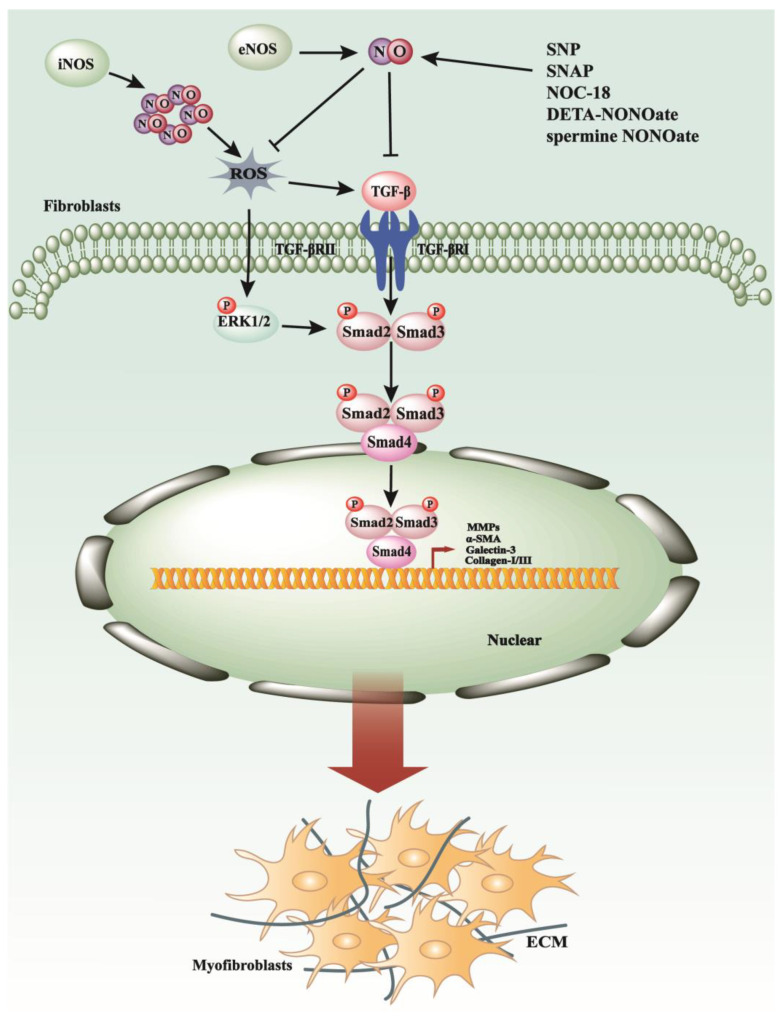
The anti-fibrosis and fibrogenesis mechanisms of NO/NOS system. Nitric oxide (NO) has demonstrated its ability to reduce reactive oxygen species (ROS) levels by forming peroxynitrite, thereby inhibiting the activation of the ERK signaling pathway and subsequently suppressing the expression of genes associated with fibrosis and inflammation. Additionally, NO can downregulate the activity of transforming growth factor-beta (TGF-β), thus attenuating its downstream signaling pathways. Within the nitric oxide synthase (NOS) system, inducible NOS (iNOS), which produces high levels of NO, is associated with pro-inflammatory effects, whereas endothelial NOS (eNOS), which generates lower levels of NO, exhibits anti-inflammatory properties.

Activated hepatic stellate cells (HSCs), which can be induced by reactive oxygen species (ROS), play a pivotal role in the process of liver fibrosis [15]. One previous study suggested that the use of the nitric oxide (NO) donor S-nitroso-N-acetyl-D,L-penicillamine (SNAP) to generate NO acts as a ROS scavenger in vitro and inhibits ERK1/2 phosphorylation, thereby reducing HSC activation [15]. Another study suggested that employing both NO donors and overexpressing endothelial NO synthase (eNOS) has been demonstrated to enhance HSC apoptosis through the generation of superoxide and hydroxyl radical intermediates, thus maintaining sinusoidal homeostasis [16]. Furthermore, sodium nitroprusside (SNP), a NO-releasing molecule, could effectively enhance mesenchymal stem cell (MSC) capacity to reduce fibrotic markers in carbon tetrachloride (CCl4)-induced liver fibrosis [17]. Therefore, exogenous NO may potentially provide an additional beneficial effect for ameliorating liver fibrosis (Figure 1).

In the liver, inducible nitric oxide synthase (iNOS) and endothelial nitric oxide synthase (eNOS) are predominant factors, while the biological actions of neuronal nitric oxide synthase (nNOS) are less understood [18]. The production of nitric oxide (NO) by eNOS plays a crucial role in maintaining liver homeostasis and preventing the development of pathological conditions in the liver [18] (Figure 1). Conversely, the upregulation of iNOS can lead to the production of large amounts of NO during the progression of fibrosis [19]. iNOS is characterized as a pro-fibrogenic mediator in high-cholesterol diet-induced liver fibrosis [20] (Figure 1). The complex and seemingly contradictory involvement of nitric oxide synthase (NOS) in the development of liver fibrosis remains poorly comprehended.

Moreover, recent studies have highlighted the diverse roles of NO in liver physiology and pathology. For instance, NO has been implicated in modulating hepatic vascular tone, regulating hepatic blood flow, and exerting anti-inflammatory effects [18]. Furthermore, the interplay between NO and other signaling pathways, such as transforming growth factor beta (TGF-β) and nuclear factor kappa B (NF-κB), contributes to the intricate pathogenesis of liver fibrosis [21,22,23].

Elucidating the precise mechanisms underlying the actions of different NOS isoforms and their downstream signaling pathways in liver fibrosis is essential for the development of targeted therapeutic interventions.

### 2.2. NO and NOS System in Pulmonary Fibrosis

Among idiopathic interstitial pneumonia, idiopathic pulmonary fibrosis (IPF) stands out as the most frequently encountered subtype, characterized by chronic and progressive fibrogenesis, primarily affecting individuals of advanced age [24]. Unfortunately, the pathogenesis of IPF still requires further exploration. Its clinical challenges are exacerbated by a propensity for misdiagnosis and the frequent yet inappropriate use of immunosuppressive therapy [24]. High mortality rates underscore the urgent need for effective management strategies, while the emergence of treatments capable of slowing disease progression offers promising opportunities for improving patient outcomes [25].

A previous study demonstrated that deficiency in all three NOS isoforms exacerbates pulmonary fibrosis in a mouse model treated with bleomycin. This was established through experiments involving wild-type mice, single-isoform NOS knockouts, and mice lacking all three NOS forms (*n/i/eNOS*^−/−^) [26]. It provides the first direct evidence that the endogenous NO and NOS systems collectively exert a crucial protective effect on the pathogenic progression of pulmonary fibrosis [26]. Interestingly, a more comprehensive study confirmed that the alveolar concentration of nitric oxide (CaNO) in individuals diagnosed with IPF was significantly higher than that observed in healthy controls, with notable correlations to various pulmonary functional parameters [27]. Researchers found that an alveolar nitric oxide concentration (CaNO) of six parts per billion (ppb) or higher was significantly associated with increased mortality, while a CaNO level of nine ppb or higher was notably correlated with accelerated disease progression in IPF patients [27]. Consequently, further investigation into the relationship between the IPF and NO is warranted. High expression of iNOS was observed in inflammatory processes, respiratory and distal lung parenchymal responses, and ECM remodeling [28] (Figure 1). Recent research suggested that thymoquinone-PLGA-PVA nanoparticles [29], amitriptyline [30], and zingerone [31] could reduce the expression of iNOS to ameliorate bleomycin-induced pulmonary fibrosis. Notably, the initial cohort in a phase 2b/3 trial investigating the effects of pulsed inhaled nitric oxide (iNO) on patients with fibrotic interstitial lung diseases has been completed successfully [32]. Patients with pulmonary fibrosis who rely on supplemental oxygen often face limitations and inconveniences that may hinder even the simplest activities of daily living [32]. The clinical trial concluded that iNO could improve physical activity among these patients, suggesting its potential as an adjunctive treatment alongside other interventions such as pulmonary rehabilitation and antifibrotic drugs [32]. Importantly, iNO therapy was found to be safe and well-tolerated [32], offering promise as a novel therapy in the future.

### 2.3. NO Regulates ECM-Degrading Proteases in Renal Fibrosis

Renal fibrosis, representing the terminal stage of various progressive renal disorders, emerges as a promising target for anti-fibrotic treatments spanning a wide spectrum of chronic kidney diseases [33,34]. It is a dynamic process characterized by intricate cellular interactions that drive fibrotic progression [35]. Renal fibrosis involves a sequence of pathological events, including injury and subsequent demise of renal parenchymal cells, infiltration by interstitial inflammatory cells, proliferation of fibroblasts, their transdifferentiation into myofibroblasts, and excessive accumulation of ECM, ultimately leading to the development of interstitial fibrosis [35].

It is widely acknowledged that mesangial cells play a pivotal role in glomerular inflammation and fibrosis [36]. Administration of NO donors, such as spermine NONOate, NOC-18, and SNAP, exerts an antiproliferative effect on mesangial cells, indicating the crucial involvement of NO in regulating the expression of profibrotic genes [36]. Furthermore, the modulation of NO production represents a promising therapeutic strategy for attenuating mesangial cell proliferation and mitigating the progression of renal fibrosis in various glomerular diseases. Additionally, exploring novel NO-based therapies may offer new avenues for the treatment of renal disorders characterized by mesangial cell dysfunction and fibrotic pathology.

Matrix metallopeptidase 9 (MMP-9) represents a pivotal enzyme within the matrix metalloproteinase family, playing a significant role in the progression of renal fibrosis. Its activity is tightly regulated at various points along the fibrotic cascade, with the tissue inhibitor of metalloproteinase-1 (TIMP-1) serving as its endogenous inhibitor to modulate MMP-9’s proteolytic function [37] (Figure 1). Elevated levels of MMPs, particularly MMP-9, induced by pro-inflammatory cytokines, have been consistently observed across various cell types, as documented in several studies [7,38,39].

A specific investigation revealed that nitric oxide (NO), produced by NO donors such as SNAP and DETA-NONOate, effectively inhibited the gelatinolytic activity of MMP-9 in rat mesangial cells (MCs) [7]. This inhibition primarily resulted from a reduction in MMP-9 mRNA expression rather than a direct blockade of the enzyme’s catalytic function [7]. Furthermore, NO has been implicated in modulating the expression of a spectrum of proteases involved in ECM degradation, along with their associated inhibitors, including MMP-9, MMP-13, plasminogen activator inhibitor-1 (PAI-1), and TIMP-1, within MCs [40,41]. Moreover, the NO donor SNAP has been shown to elevate TIMP-1 levels, with this up-regulation mediated through a TGF-β-dependent mechanism in these cells [42] (Figure 1). Meanwhile, in the unilateral ureteral obstruction (UUO) model, extra intake of L-arginine could increase the synthesis of NO and the expressions of VEGF and eNOS, reduce HIF-1α and TGF-β1 expressions, and ultimately reverse renal fibrosis [43] (Figure 1). In the same model, a recent paper indicated that a NO donor, the iron nitrosyl complex DNIC, into pH-responsive NPs, represents a therapeutic potential to ameliorate renal fibrosis [44]. Consequently, NO donors hold promise as pharmacological agents for attenuating fibrosis. However, further investigation is warranted to elucidate the underlying mechanisms governing their therapeutic effects.

## 3. The Axis of HO/CO and Exogenous CO in Attenuation of Fibrosis

Carbon monoxide (CO) is a colorless, odorless gas that is slightly less dense than air. It is highly toxic to humans and animals due to its ability to bind with hemoglobin in the blood, reducing its capacity to transport oxygen. CO is produced by incomplete combustion of carbon-containing fuels and is a component of vehicle exhaust and cigarette smoke. Despite its toxicity, carbon monoxide has some industrial applications, such as in the production of metal carbonyls and as a reducing agent in metallurgy [45]. However, as for one of the gasotransmitters, endogenous CO is a biologically active molecule in states of health and disease and is produced when heme is degraded by heme oxygenase (HO), including inducible isoform HO-1 and constitutive isoform HO-2, along with free iron and biliverdin as other products [46,47]. HO-2 plays a critical role in neurotransmission and the regulation of vascular tone and is typically found in the brain, liver, and endothelium [48]. Conversely, an upregulation of HO-1 in tissues signals disturbed cellular homeostasis [49]. Consequently, the physiological levels of CO produced through HO-1 induction serve as a restorative signal within the human body, aiming to reestablish intracellular, neural, and vascular functions [50]. It is believed that the HO-1/CO pathway offers cytoprotection in animal models suffering from organ injury and disease by modulating inflammation and apoptosis [51].

In recent years, both endogenously produced carbon monoxide (CO) and low concentrations of exogenously administered CO have been recognized for their multifaceted cytoprotective roles [52]. These include anti-apoptotic, anti-inflammatory effects, vasomodulation, preservation of homeostasis, induction of preconditioning, and modulation of cell differentiation [52]. The last decade has seen the establishment of the therapeutic potential of CO through inhalation therapy [53]. However, it is crucial to note that unregulated and prolonged inhalation may result in CO toxicity and, consequently, fatal outcomes in patients [53]. Excitingly, recent advancements have unearthed the therapeutic promise of CO via the advent of innovative carbon monoxide-releasing molecules (CORMs) [53,54]. These CORMs are engineered to facilitate the safe administration of CO and present a viable alternative to inhalation therapy [53]. A diverse array of CORMs has been synthesized, featuring central transition metals such as iron (Fe), manganese (Mn), and cobalt (Co), with CO acting as a coordinating ligand [53,54]. The liberation of CO from these complexes necessitates specific conditions or ligand interactions [53,54]. Notably, CORM-1 (Mn_2_CO_10_), CORM-2 ((Ru (CO)_3_Cl_2_)_2_), and CORM-3 (Ru (CO)_3_Cl-glycinate) have undergone extensive research for their therapeutic potential [55,56]. They have been demonstrated to release CO under physiological conditions, thereby eliciting their respective beneficial effects [55,56].

Concurrent with advancements in CO donors and their proven biological benefits, there has been a growing interest in the potential anti-fibrotic effects of CO on various organs. An increasing body of literature now reports that CO may play a significant role in mitigating fibrotic diseases.

### 3.1. CO Inhibits the Proliferation and Activation of Fibroblasts in Pulmonary Fibrosis

**Figure 2:**
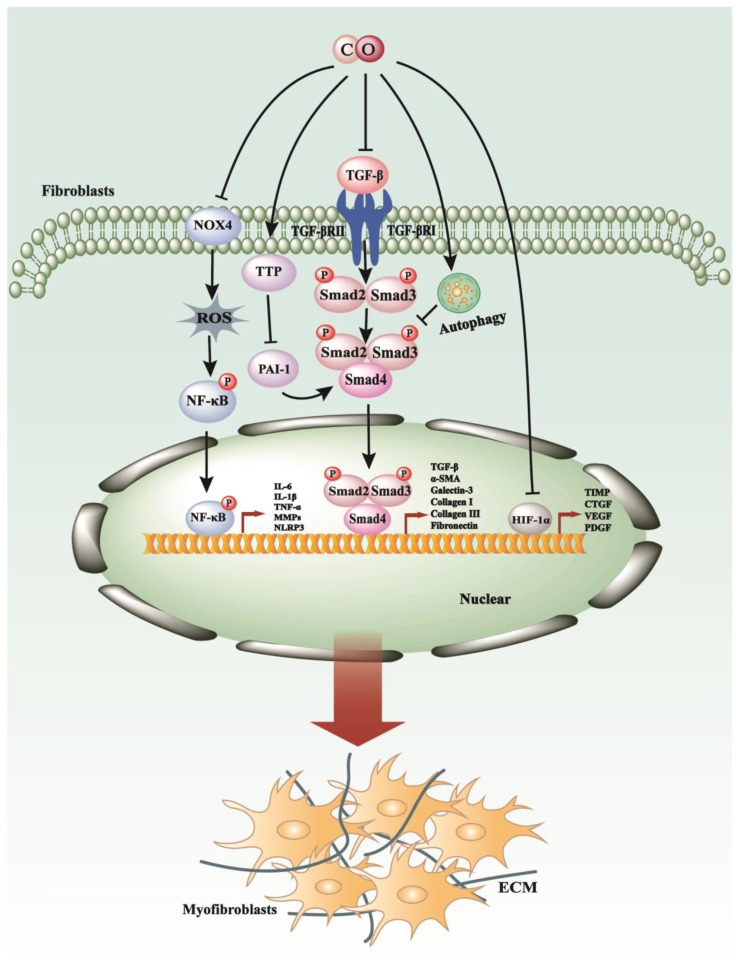
The anti-fibrosis mechanisms of CO. Low doses of both exogenous and endogenous CO can modulate the TGF-β, NF-κB, HIF-1*α* signaling pathways by down-regulating the expression of genes related to fibrosis and inflammation. Furthermore, CO is capable of inhibiting TGF-β signaling through the stimulation of autophagic processes. Concurrently, CO also inhibits fibroblast proliferation, thereby contributing to the reversal of fibrogenesis.

A study has demonstrated that administration of a low concentration of exogenous CO, at 250 ppm, to mice exhibits the potential for halting the progression of pulmonary fibrosis in vivo [57]. This inhalation of CO concurrently resulted in an upregulation of p21Cip1 expression and downregulation of cyclin A and D levels, thereby inhibiting fibroblast proliferation [57] (Figure 2). Moreover, it may diminish fibronectin (FN) and collagen-1 levels by modulating the inhibitor of DNA binding 1 (Id1) in vitro [57]. Additionally, CO was observed to suppress α–SMA expression while enhancing small proline-rich protein-1a expression (Sprr1a), although the correlation between fibrosis and Sprr1a remains unclear.

Another study illustrated that CO-bound hemoglobin vesicles (CO-HbV), a type of nanotechnology-based CO donor, could mitigate IPF by suppressing NOX4 activity to reduce reactive oxygen species (ROS) and decreasing transforming growth factor-β (TGF-β) and inflammatory cytokines including TNF-α, IL-1β, and IL-6 in the lung [46] (Figure 2). Furthermore, a recent study reported that the administration of the slow-releasing carbon monoxide-releasing molecule CORM-A1 at a dosage of 5 mg/kg was capable of reducing the expression of pro-fibrogenic cytokines such as COX-2, TNF-α, and α-SMA, as well as serum hydroxyproline levels, thereby alleviating paraquat-induced pulmonary fibrosis [58]. Cellular senescence, the irreversible progression of cell division induced by various physiological and environmental stressors, is recognized to contribute to fibrogenesis [59]. Previous research from our laboratory has suggested that CORM-A1 may attenuate bleomycin-induced senescence in pulmonary fibroblasts by facilitating the formation of stress granules to sequestrate PAI-1 [8]. Notably, a clinical trial has demonstrated that low-dose CO gas inhalation is well tolerated and deemed safe for administration to patients with IPF in phase II clinical trials [60]. In conclusion, low-dose CO exhibits anti-fibrotic effects on the lung and holds promise as a potential therapy to ameliorate pulmonary fibrosis.

### 3.2. CO Regulates MKK3, ERK-MAPK, and TGF-β/Smad Pathways in Renal Fibrosis

In 2006, the introduction of inhaled low-dose CO was initially proposed as a therapeutic approach to counteract the chronic fibroinflammatory changes associated with chronic allograft nephropathy (CAN), aiming to enhance long-term renal allograft function and improve transplant outcomes [61]. Subsequently, a study demonstrated that the administration of low-dose CO inhibits renal fibrosis induced by unilateral ureteral obstruction via the MKK3 pathway [62]. Following these findings, another study revealed complementary evidence indicating that low-dose CO could suppress the expression of TGF-β/Smad and ERK-MAPK pathways, thereby mitigating the fibro-inflammatory processes of CAN and ameliorating renal allograft function [63] (Figure 2).

The aforementioned data suggest that the administration of CO at low concentrations may confer nephroprotective effects, particularly by attenuating the progression of renal fibrosis. This therapeutic strategy holds promise in the management of chronic kidney diseases characterized by fibrotic changes, presenting a potential avenue for improving renal outcomes in transplant recipients. Further investigations into the mechanisms underlying CO-mediated nephroprotection and its clinical applications are warranted to fully elucidate its therapeutic potential in renal fibrosis and related conditions.

### 3.3. CO Modulates TGF-β Pathways and Autophagy in Myocardial Fibrosis

Following human immunodeficiency virus (HIV) infection, the administration of the protease inhibitor ritonavir has been documented to precipitate cardiac dysfunction, clinically manifested as pathological fibrosis [64]. Emerging research delineates that inhalation of carbon monoxide at a low concentration (250 ppm) can mitigate ritonavir-induced myocardial fibrosis through modulation of both canonical (Smad2-mediated) and non-canonical (p38 MAP kinase-mediated) TGF-β1 signaling pathways [64]. Concurrently, CO administration induces a phenotypic shift in cardiac macrophage populations from the M1 pro-inflammatory to the M2c anti-inflammatory subset, a transition concomitant with CO-induced autophagy [64] (Figure 2). Despite these findings, the comprehensive anti-fibrotic mechanisms by which CO modulates TGF-β signaling and induces autophagy remain to be fully elucidated.

### 3.4. CO Attenuates Inflammation and Aging in Liver Fibrosis

Metabolic-associated fatty liver disease (MAFLD) encompasses a range of chronic liver conditions, progressing from steatohepatitis to advanced hepatic fibrosis, cirrhosis, and, ultimately, hepatocellular carcinoma [65]. Recent research has unveiled that a nanomicellar carbon monoxide-releasing molecule, SMA/CORM2, can mitigate liver fibrosis induced by a high-fat methionine- and choline-deficient (HF-MCD) diet by inhibiting the hypoxia-inducible factor 1-alpha (HIF-1α)-mediated inflammatory cascade [65] (Figure 2).

Within the realm of non-alcoholic fatty liver disease (NAFLD), significant fibrosis serves as a critical prognostic marker, correlating closely with disease progression and outcomes [66]. Our previous investigation into the mechanism of action between carbon monoxide (CO) and liver fibrosis demonstrated that CORM2 attenuates acetaminophen-induced liver injury by upregulating hepatic HO-1 and Parkin expression. Furthermore, we identified tristetraprolin (TTP) as a potential mechanistic focal point for anti-inflammatory compounds, with CO inducing increased TTP expression [67,68].

Additionally, CO has emerged as a novel inducer of stress granules by activating the integrated stress response [69]. Our study indicates that carbon monoxide-dependent activation of TTP leads to a reduction in plasminogen activator inhibitor-1 (PAI-1) levels within stress granules (SGs), ultimately ameliorating age-related NAFLD [70] (Figure 2).

Expanding on these findings, further exploration into the interplay between carbon monoxide and various cellular pathways involved in liver fibrosis could provide insights into potential therapeutic strategies for MAFLD and NAFLD, offering new avenues for clinical intervention in this increasingly prevalent liver fibrosis.

## 4. H_2_S Exerts Suppressive Impacts on the Process of Fibrosis

Hydrogen sulfide (H_2_S) is a colorless, flammable gas with a characteristic foul odor reminiscent of rotten eggs. It is slightly soluble in water and is denser than air. H_2_S is highly toxic to humans and animals, affecting the respiratory and central nervous systems. Hydrogen sulfide is produced naturally through the decay of organic matter and is also a byproduct of industrial processes such as petroleum refining and wastewater treatment. Despite its toxicity, hydrogen sulfide has some industrial applications, including in the production of sulfur and in the synthesis of various organic compounds [71]. The endogenous form of H_2_S is synthesized in mammalian cells, predominantly within the cytoplasm and mitochondria, via enzymatic catalysis involving L-cysteine and D-cysteine substrates. Key enzymes involved in this biosynthetic pathway include cystathionine-β-synthase (CBS), cystathionine-γ-lyase (CSE), 3-mercaptopyruvate sulfurtransferase (3-MST), and D-amino acid oxidase (DAO) [72,73,74]. Externally, hydrogen sulfide (H_2_S) can also be obtained from various donor compounds, such as sodium hydrosulfide (NaHS); sodium sulfide (Na_2_S); a variety of sulfur-containing molecules like S-allyl-cysteine (SAC); novel H_2_S-releasing compounds like GYY4137, AP39, and AP123; as well as SG1002, S-propargyl cysteine, and agents like sodium thiosulfate. Moreover, sulfurous mineral water and natural products such as garlic-derived polysulfide, diallyl disulfide, and diallyl sulfide also contribute to the exogenous delivery of H_2_S [75]. Over the past few decades, the protective properties of H_2_S at low concentrations have become increasingly evident. These benefits primarily stem from the molecule’s inherent anti-inflammatory, antioxidant, and anti-fibrotic properties, which apply to both exogenously administered and endogenously produced H_2_S [76].

### 4.1. H_2_S Regulates TGF-β Pathways, Inflammation, Autophagy, and Oxidative Stress in Renal Fibrosis

The presence of hydrogen sulfide (H_2_S) in rat kidneys was initially documented in the early 1980s [77]. Subsequent research has confirmed the prolific expression of enzymes responsible for H_2_S synthesis within renal tissues [78,79]. Specifically, cystathionine-β-synthase (CBS) has been identified as the predominant enzyme for H_2_S production in the kidney, primarily localized in the proximal renal tubules [80,81]. Conversely, cystathionine-γ-lyase (CSE) is present in lesser quantities, with its distribution primarily observed in the renal glomeruli, interstitium, and interlobular arterioles. Moreover, 3-mercaptopyruvate sulfurtransferase (3-MST) is chiefly found within the proximal tubular epithelium [81,82].

**Figure 3:**
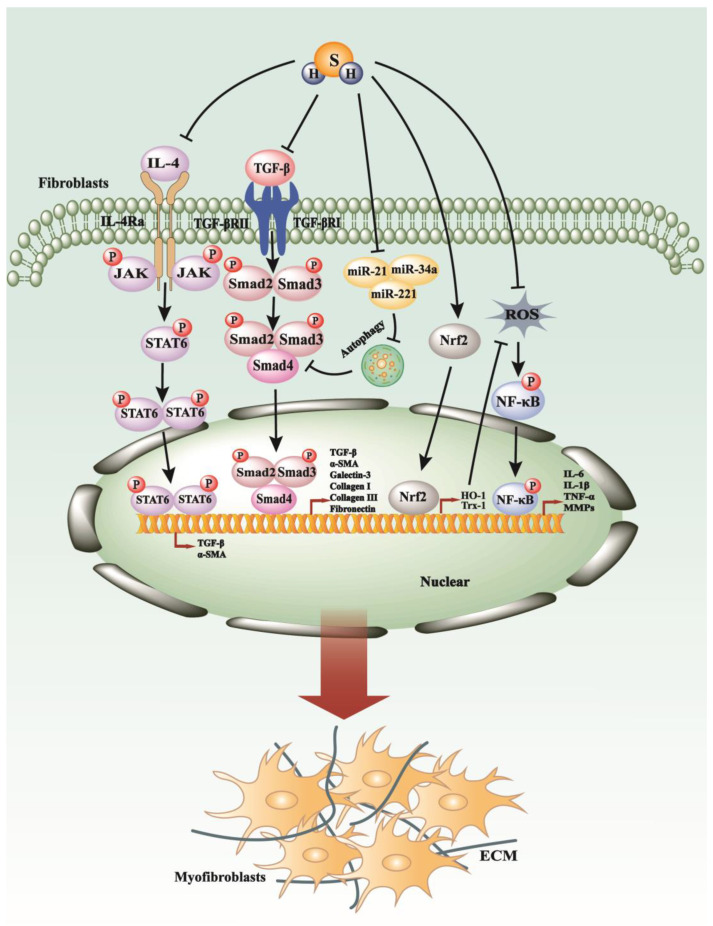
The anti-fibrosis mechanisms of H_2_S. H_2_S can efficaciously decelerate the progression of fibrosis by modulating key signaling pathways, including NF-κB, TGF-β, IL-4/STAT6, and by attenuating ROS production via the up-regulation of Nrf2.

Over the last decade, research emphasis on H_2_S has primarily focused on its role in inducing renal fibrosis through unilateral ureteral obstruction (UUO) and diabetes mellitus. In studies utilizing mouse models of kidney fibrosis induced by UUO, treatment with NaHS has been shown to suppress the levels of TGF-β1 and the activation of Smad3 and NF-κB, thereby improving kidney fibrosis [83]. Further investigation into the renal protective mechanisms of hydrogen sulfide using NaHS in a rat model of UUO revealed inhibition of cellular proliferation, diminished DNA synthesis, and attenuation of the expression of proteins associated with proliferation, such as proliferating cell nuclear antigen and c-Myc. Additionally, NaHS prevented the transformation of renal fibroblasts into myofibroblasts by inhibiting the TGF-β1-Smad and MAPK signaling pathways [81]. Moreover, H_2_S therapy in UUO-induced renal fibrosis suppressed both M1 and M2 macrophage infiltration and NLRP3 inflammasome activation, thereby attenuating downstream NF-κB and IL-4/STAT6 signaling cascades [84] (Figure 3). Recently, a study demonstrated that NaHS treatment greatly improved chronic kidney disease (CKD) characterized by hypoxia conditions, restored ten-eleven translocation (TET) activity, reduced DNA methylation, and increased Klotho expression [85].

In a diabetic rat model characterized by renal fibrosis, H_2_S has been observed to potentially mitigate the progression of fibrosis [86]. This amelioration may be attributed to H_2_S’s ability to up-regulate the expression of connexins such as CX40, CX43, and CX45, which play a role in cell communication and tissue homeostasis [86]. Additionally, H_2_S appears to influence the balance of matrix metalloproteinases (MMPs) and tissue inhibitors of metalloproteinases (TIMPs), critical regulators of extracellular matrix turnover and fibrotic processes [86]. The therapeutic actions of H_2_S in diabetic renal fibrosis may include the inhibition of autophagy pathways, upregulation of superoxide dismutase (SOD), and downregulation of signaling molecules such as AKT, TGF-β1, and NF-κB [87] (Figure 3). Furthermore, it has been hypothesized that H_2_S modulates diabetic renal fibrosis by influencing TGF-β1 pathways, thereby adjusting the equilibrium between mediators that contribute to fibrogenesis and those that counteract it [88].

Collectively, these studies have primarily focused on TGF-β1 signaling, inflammation, and oxidative stress, irrespective of whether renal fibrosis was induced by UUO or diabetes. Further exploration of H_2_S’s therapeutic potential in renal fibrosis may provide valuable insights into novel treatment strategies for kidney diseases.

### 4.2. H_2_S Modulates PI3K/AKT1, JAK/STAT, PKC-ERK1/2MAPK Pathways in Myocardial Fibrosis

Recent studies have increasingly recognized that diminished levels of hydrogen sulfide (H_2_S) within the myocardium are significantly associated with the severity of myocardial fibrosis [89,90,91]. This association implicates H_2_S as a potentially important modulator of fibrotic processes in cardiac tissue, where its decline might contribute to the progression and severity of fibrotic pathology in the heart [89,90,91]. Consequently, many researchers have begun to explore the use of exogenous H_2_S to ameliorate myocardial fibrosis. Previous investigations have unveiled the potent role of H_2_S in mitigating the development of cardiac hypertrophy and fibrosis induced by excessive hemodynamic pressure [92,93]. Furthermore, the application of H_2_S has been demonstrated to alleviate the pathological remodeling and functional deterioration of the left ventricle associated with heart failure [92,93]. The therapeutic potential of H_2_S in these contexts is believed to be linked to its promotion of a proangiogenic environment, which facilitates vascular growth and, hence, enhances cardiac tissue viability [92,93]. Subsequently, the biological mechanisms of H_2_S concerning cardiac fibrosis have been gradually elucidated. Recent investigations have revealed the following findings: H_2_S can downregulate MMP, TIMP, and TGF-β1 expression to attenuate cardiac fibrosis [94]. Additionally, H_2_S may confer cardioprotective effects on left ventricular remodeling observed in smoking rats by reducing oxidative stress through PI3K/Akt-dependent activation of Nrf2 signaling [95]. H_2_S has been identified as a potential therapeutic agent for myocardial fibrosis, exerting its effects through the attenuation of myocardial autophagy and the activation of the PI3K/AKT1 signaling pathway [96].

H_2_S has demonstrated promising potential for mitigating the detrimental impacts of chronic alcohol consumption on the myocardium [97]. This is achieved through the inhibition of collagen deposition and the reduction in the expression of autophagy-related proteins such as Beclin 1, Atg3, and Atg7, along with fibrosis-related proteins including MMP8, MMP13, MMP14, MMP17, and TGF-β1 [97]. Additionally, H_2_S has been observed to downregulate the expression of the microRNAs miR-21 and miR-221, which are implicated in the pathogenesis of myocardial fibrosis [97]. Moreover, H_2_S has shown promise in attenuating myocardial fibrosis in diabetic rat models. Its beneficial effects are linked to the downregulation of the JAK/STAT signaling pathway, which in turn suppresses oxidative stress, endoplasmic reticulum (ER) stress, inflammation, and cellular apoptosis [98]. Furthermore, H_2_S is believed to counteract thyroxine-induced myocardial fibrosis by inducing autophagy, mediated by increased activity of the PI3K/AKT signaling axis and the simultaneous downregulation of microRNAs miR-21, miR-34a, and miR-214 [99] (Figure 3).

Administration of exogenous H_2_S has also shown efficacy in reducing myocardial fibrosis by potentially modulating the PKC-ERK1/2 MAPK signaling pathway. This modulation rectifies imbalances in the expression of MMPs and TIMPs, thus dampening inflammatory responses [100]. Additionally, H_2_S has demonstrated a protective effect against streptozotocin-induced myocardial fibrosis in diabetic rodent models by downregulating both the canonical Wnt signaling pathway and the TGF-β1/Smad3 axis, resulting in reduced myocardial collagen deposition [101]. In addition, H_2_S has been shown to attenuate myocardial fibrosis induced by doxorubicin by inhibiting excessive ER stress and autophagy through upregulation of the PI3K/AKT/mTOR signaling pathway [102]. Exogenous H_2_S has been found to ameliorate myocardial cell fibrosis by upregulating the expression of microRNA miR-133a and connective tissue growth factor (CTGF) [103]. Additionally, H_2_S administration inhibits necroptosis, thereby mitigating the hypoxia-induced proliferation of cardiac fibroblasts through the activation of sirtuin 3 (SIRT3) [104].

Overall, the beneficial effects of exogenous H_2_S mainly involve the suppression of myocardial collagen deposition, ER stress, inflammation, autophagy, and oxidative stress in myocardial fibrosis. Further research into the precise mechanisms and clinical applications of H_2_S in treating myocardial fibrosis holds promise for developing novel therapeutic interventions in cardiac pathology.

### 4.3. H_2_S Regulates the Proliferation and Activation of HSCs in Liver Fibrosis

Diminished H_2_S production and cystathionine γ-lyase (CSE) expression have been associated with increased intrahepatic resistance and the development of portal hypertension in a carbon tetrachloride (CCl4)-induced rat model of liver cirrhosis [105]. Researchers have explored the therapeutic potential of exogenous H_2_S to supplement endogenous levels in this context. Recent studies indicated that the administration of exogenous H_2_S can mitigate CCl4-induced hepatotoxicity, liver cirrhosis, and portal hypertension through antioxidative, anti-inflammatory, cytoprotective, and antifibrotic mechanisms [106]. Furthermore, exogenous H_2_S has been observed to inhibit the proliferation of activated hepatic stellate cells (HSCs), leading to cell cycle arrest and apoptosis, thus alleviating hepatic fibrosis and ECM expression [9]. It also reduces hepatic expression of Angiotensin II Receptor 1 (AGTR1), potentially slowing down the progression of hepatic fibrosis [107]. However, a recent study has presented contrasting findings, suggesting that both exogenous and endogenous H_2_S might promote the proliferation and activation of HSCs, while H_2_S suppression could have the opposite effect, with these outcomes possibly mediated by changes in cellular bioenergetics [108].

Given these conflicting results, further research using more comprehensive animal models and cellular studies is essential to clarifying the purported protective role of H_2_S and its underlying molecular mechanisms in liver fibrosis. This will contribute to a better understanding of the complex biological interactions involving H_2_S in liver pathology.

### 4.4. H_2_S Restrained the Inflammation and Oxidative Stress in Pulmonary Fibrosis

Both the lung and pulmonary arteries are rich in active cystathionine γ-lyase (CSE) protein, enabling endogenous hydrogen sulfide (H_2_S) production [109]. Numerous studies have indicated that exogenous H_2_S may play a protective role in reversing the progression of pulmonary fibrosis. One study suggested that exogenous H_2_S could attenuate pulmonary fibrosis by reducing oxidative stress [110] (Figure 3). Furthermore, H_2_S has been shown to mitigate the progression of epithelial-to-mesenchymal transition (EMT) in alveolar epithelial cells by inhibiting Smad2/3 phosphorylation [111].

Emerging research has revealed the therapeutic potential of H_2_S in treating bleomycin (BLM)-induced pulmonary fibrosis in male rat models [112]. It has been postulated that H_2_S exerts its protective effects in BLM-induced pulmonary fibrosis by suppressing the expression of the NF-κB p65 subunit and modulating the Th1/Th2 cytokine profile [112]. Moreover, administration of the H_2_S donor sodium hydrosulfide (NaHS) has been associated with the nuclear accumulation of nuclear factor erythroid 2-related factor 2 (Nrf2) within lung tissue, leading to upregulation of antioxidant genes such as heme oxygenase-1 (HO-1) and thioredoxin-1 (Trx-1), thereby conferring resistance to fibrotic changes in the lungs of rats exposed to cigarette smoke [113] (Figure 3). Additionally, H_2_S has been found to mitigate inflammation induced by cigarette smoke through the inhibition of phosphorylation of extracellular signal-regulated kinase 1/2 (ERK1/2), c-Jun N-terminal kinase (JNK), and p38 mitogen-activated protein kinases (MAPKs), as well as the activation of NF-κB [113].

These findings underscore the multifaceted actions of H_2_S and emphasize the complexity of its role in pulmonary fibrosis, particularly concerning the potential consequences of excessive administration. Further research is warranted to elucidate the precise mechanisms underlying the therapeutic effects of H_2_S in pulmonary fibrosis and to assess its safety and efficacy in clinical settings.

## 5. The Novel Gasotransmitter H_2_ in the Process of Fibrosis

Hydrogen (H_2_) is a colorless, odorless, and tasteless diatomic gas with the lightest known density. It is highly flammable and burns in the air with an almost invisible flame. H_2_ is the most abundant element in the universe and is commonly found in water, hydrocarbons, and organic matter. Hydrogen gas is used extensively in various industrial processes, including in the production of ammonia and methanol, as well as in hydrogenation reactions. Additionally, it holds promise as a clean energy carrier in fuel cells for transportation and power generation, offering a potential solution to environmental concerns related to carbon emissions. The regression of skin tumors induced by hydrogen was initially reported in 1975 [114]. However, prior to 1975, molecular hydrogen was generally considered inert in mammalian cells, with documented activity limited to certain bacterial species [115]. In recent years, the field of hydrogen research has experienced significant expansion, with numerous scholars actively investigating its properties and potential benefits. The underlying mechanisms of H_2_ action have been progressively elucidated, encompassing anti-oxidative, anti-inflammatory, and regulation of cell death pathways. Additionally, to harness the therapeutic potential of hydrogen, two main administration methods have emerged: hydrogen gas and hydrogen water.

The clinical application of hydrogen gas is impeded by its inherent flammability risk and potential for explosion, particularly at concentrations exceeding 4.7% in the air, which renders its safe use in therapeutic settings challenging [116]. Consequently, researchers are exploring various alternative forms of hydrogen administration to explore its therapeutic efficacy.

### 5.1. H_2_ Regulates TGF-β1 Pathways, Oxidative Stress, and Inflammatory Response in Pulmonary Fibrosis

**Figure 4:**
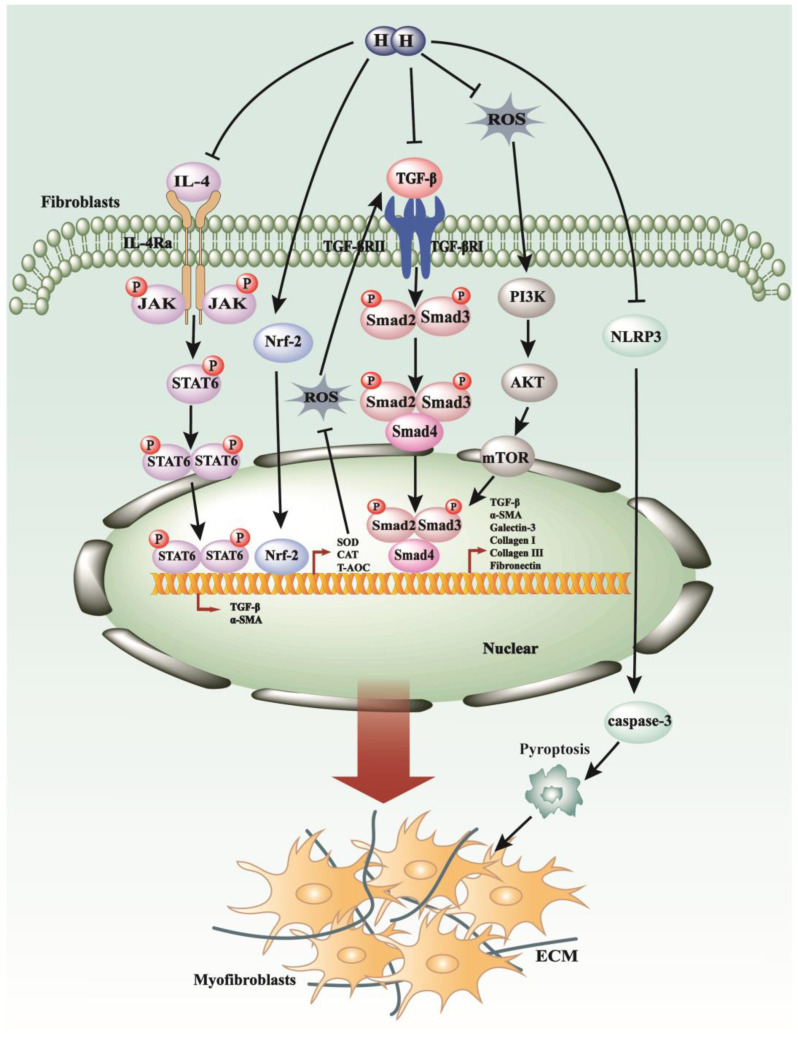
The anti-fibrosis mechanisms of H_2_ in fibrotic diseases. H_2_ can decelerate fibrotic process modulation by targeting key signaling cascades such as JAK/STAT pathways, TGF-β/Smads pathways, and PI3K/AKT/mTOR pathways, in addition to diminishing ROS production through the up-regulation of Nrf2. Additionally, molecular hydrogen is capable of inhibiting the assembly of the NLRP3 inflammasome and reducing the release of inflammatory mediators, thereby exerting control over the initiation and progression of fibrotic conditions.

Lipopolysaccharide (LPS)-induced pulmonary fibrosis is frequently utilized as a model to induce acute lung injury and acute respiratory distress syndrome (ARDS) for investigational purposes. Studies have demonstrated that pulmonary oxidative stress and epithelial-to-mesenchymal transition (EMT) induced by LPS can be mitigated by intra-peritoneal administration of hydrogen-rich saline [117]. Researchers noted a significant reduction in malondialdehyde (MDA) levels and restoration in antioxidative biomarkers such as total antioxidant capacity (T-AOC), catalase (CAT), and superoxide dismutase (SOD) activity following treatment with hydrogen-rich saline [117] (Figure 4). Additionally, the diminished expression of E-cadherin and increased expression of α-SMA in pulmonary tissues induced by LPS were markedly attenuated by hydrogen-rich saline administration [117]. Furthermore, hydrogen water administration enhanced Nrf2 expression in human lung fibroblasts, ameliorating paraquat-induced acute lung injury and promoting antioxidant production to exert its antioxidative effects [118] (Figure 4). These examples highlight the potential therapeutic benefits of hydrogen-rich water.

Hydrogen inhalation therapy provides a direct means of delivering hydrogen molecules to the lungs. Inhalation of hydrogen gas has been shown to inhibit epithelial-to-mesenchymal transition (EMT) in bleomycin (BLM)-induced idiopathic pulmonary fibrosis by decreasing TGF-β1 levels [119] (Figure 4). Moreover, hydrogen inhalation therapy can suppress the elevation of pro-inflammatory cytokines such as IL-6, IL-4, and IL-13 in bleomycin-induced lung injury, thereby inhibiting M2-biased macrophage polarization and reducing alveolar fibrosis [120].

Collectively, these insights underscore the multifaceted therapeutic potential of hydrogen-based interventions in mitigating lung injury, fibrosis, and inflammation, thereby paving the way for novel treatment modalities in respiratory medicine.

### 5.2. H_2_ Modulates the p38 MAPK, JAK/STAT Pathways, Pyroptosis, and Oxidative Stress in Myocardial Fibrosis

A majority of cardiovascular diseases inevitably progress to heart failure, characterized by cardiac fibrosis, a condition marked by the excessive deposition of ECM components within myocardial tissue [121]. This prevalent pathology presents a significant clinical challenge [122]. Initial investigations into therapeutic interventions targeting cardiac fibrosis have revealed promising outcomes with the use of hydrogen-containing saline (HCS). Treatment with HCS has been associated with enhanced cardiac function and attenuation of fibrosis. Notably, these effects are accompanied by reductions in reactive oxygen species (ROS) levels, decreased phosphorylation of p38 MAPK and Smad2/3, and suppression of TGF-β1 and connective tissue growth factor (CTGF) expression [123]. Moreover, pathological cardiac hypertrophy often involves interstitial and perivascular fibrosis. Hydrogen-rich saline (HRS) has demonstrated a protective effect against inflammation, as evidenced by its ability to ameliorate pressure overload-induced cardiac hypertrophy in rats by downregulating the Janus kinase-signal transducer and activator of transcription (JAK-STAT) signaling pathway [124] (Figure 4). In a novel approach, inhalation of hydrogen gas has shown promise in mitigating cardiomyocyte damage induced by hypoxia and restraining the migration and activation of cardiac fibroblasts stimulated by angiotensin II [125]. These protective effects are primarily attributed to the inhibition of the NLR family pyrin domain containing 3 (NLRP3)-mediated pyroptosis pathway, a form of programmed cell death associated with inflammation [125] (Figure 4). Furthermore, studies have elucidated that HRS exerts antioxidative effects and reduces myocardial collagen content by inhibiting the TGF-β signaling pathway, thereby attenuating myocardial fibrosis in spontaneously hypertensive rats [126] (Figure 4).

These findings highlight the diverse therapeutic possibilities of hydrogen-based treatments in alleviating cardiac fibrosis and its related conditions, presenting innovative approaches for managing cardiovascular ailments.

### 5.3. H_2_ Exerts a Suppressive Effect on the Development of Liver Fibrosis

Hydrogen-rich saline has exhibited efficacy in facilitating the recovery from acute liver injury and hepatic cirrhosis while also promoting hepatocyte proliferation by mitigating reactive oxygen species (ROS) levels [116]. Simultaneously, it suppresses pro-apoptotic factors such as c-Jun N-terminal kinase (JNK) and caspase-3 [116]. Non-alcoholic fatty liver disease (NAFLD), a condition with potential progression to hepatic fibrosis, has shown responsiveness to treatment with hydrogen-rich saline [127]. A specific study demonstrated notable improvements in NAFLD, possibly attributed to the reduction in oxidative stress and the upregulation of hepatic peroxisome proliferator-activated receptors (PPAR)α and PPARγ [127]. Furthermore, one study demonstrated that hydrogen-rich water could reverse hepatocyte apoptosis and alleviate hepatic inflammation and fibrosis through an HO-1/IL-10-independent pathway [128].

These findings not only emphasize the therapeutic potential of hydrogen-based interventions in liver diseases but also shed light on the intricate molecular mechanisms underlying their beneficial effects, providing valuable insights for the development of novel therapeutic strategies.

### 5.4. H_2_ Regulates the Development of EMT and Oxidative Stress in Renal Fibrosis

Previous studies have demonstrated that treatment with hydrogen-rich saline significantly mitigates fibrosis and diminishes macrophage infiltration in UUO-affected kidneys [129,130]. These effects are attributed to the reduction in malondialdehyde (MDA) levels, indicative of lipid peroxidation, and the enhancement of superoxide dismutase (SOD) activity, a pivotal antioxidant defense mechanism [130]. Moreover, hydrogen-rich water has been shown to attenuate renal fibrosis and suppress the epithelial–mesenchymal transition (EMT) process in human kidney proximal tubular epithelial cells (HK-2 cells) by upregulating Sirt1, a molecule modulated by transforming growth factor beta 1 (TGF-β1) [10]. These findings underscore the potential of hydrogen-based therapies in combating renal fibrosis and offer insights into the underlying molecular mechanisms, thereby presenting promising avenues for the management of kidney diseases.

### 5.5. H_2_ Plays a Suppressive Role in Peritoneal Fibrosis by Targeting TGF-β/PAI-1, Oxidative Stress, and Inflammation Pathways

Postsurgical peritoneal adhesions (PPAs), characterized by the adherence of viscera to the abdominal wall, can lead to the formation of pathological fibrous structures within the peritoneal cavity. Hydrogen-rich saline has shown promise in ameliorating PPAs, potentially through the inhibition of TGF-β1 and plasminogen activator inhibitor-1 (PAI-1), augmentation of tissue plasminogen activator (t-PA), and reduction in oxidative stress and inflammation [131]. Peritoneal dialysis (PD) is recognized as a viable therapeutic modality for managing complications associated with end-stage renal disease (ESRD) [132]. However, continuous exposure to biologically incompatible peritoneal dialysis solutions has been implicated in the development of peritoneal fibrosis [133]. Recent studies suggest that the introduction of hydrogen-enriched peritoneal dialysate mitigates the extent of fibrotic changes within the peritoneal membrane [133]. This effect is attributed to the anti-fibrogenic properties of molecular hydrogen, which operate through the modulation of the oxidative-stress-associated ROS/PTEN/AKT/mTOR signaling pathway [133] (Figure 4).

These findings suggest the potential of hydrogen-based strategies for addressing peritoneal fibrosis, offering valuable insights into the underlying mechanisms. This opens up promising avenues for refining peritoneal dialysis approaches, thereby potentially improving outcomes for individuals managing end-stage renal disease (ESRD).

## 6. SO_2_ in the Process of Myocardial Fibrosis and Other Cardiovascular Diseases

**Figure 5:**
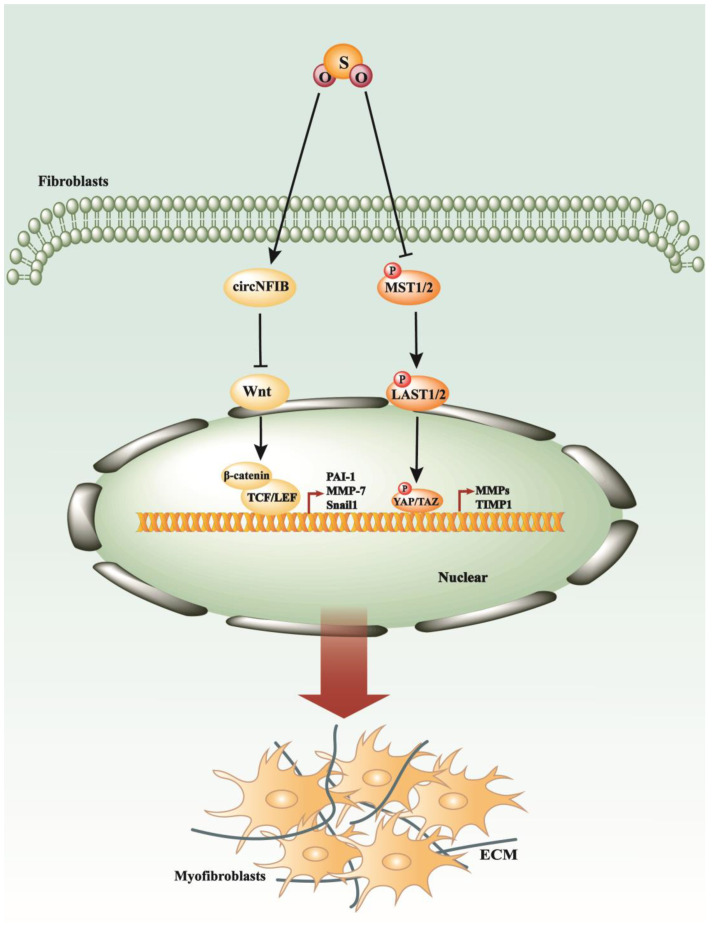
SO_2_ exerts its antifibrotic effects by modulating key intracellular signaling cascades. SO_2_ can effectively inhibit the progression of fibrotic processes by suppressing critical signaling pathways, including Wnt/β-catenin and Hippo–MST.

Sulfur dioxide (SO_2_) is a colorless gas with a pungent odor. It is slightly soluble in water, forming sulfurous acid (H_2_SO_3_), and is denser than air. Sulfur dioxide is produced primarily by the combustion of sulfur-containing fuels, such as coal and oil, and by the roasting of metal sulfide ores. Due to its acidic nature, sulfur dioxide is a major contributor to acid rain formation when it reacts with atmospheric water vapor. It also plays a role in atmospheric chemistry, participating in the formation of sulfate aerosols, which can affect climate and air quality. Industrially, sulfur dioxide is used in the production of sulfuric acid, as a preservative in food and beverages, and as a bleaching agent in the pulp and paper industry [134]. Recent studies have unveiled an intrinsic SO_2_-generating system within the cardiovascular system [135]. In mammals, endogenous generation of sulfur dioxide (SO_2_) occurs through the metabolism of the sulfur-containing amino acid L-cysteine [136]. Analogous to other gasotransmitters, SO_2_ possesses several physiological advantages, including vasodilatory effects, anti-inflammatory actions, and the promotion of vascular collagen remodeling [137]. A specific study has outlined the effectiveness of SO_2_ in mitigating myocardial fibrosis in diabetic murine models, suggesting that the underlying mechanism involves the attenuation of apoptosis and endoplasmic reticulum stress (ERS) through the suppression of the Hippo–MST signaling pathway [109] (Figure 5). Additionally, it has been demonstrated that endogenous SO_2_ enhances the expression of circNFIB, a circular RNA, which in turn inhibits the Wnt/β-catenin and p38 MAPK signaling cascades, thus alleviating the progression of myocardial fibrosis [138] (Figure 5). Another study revealed that endogenous SO_2_ might inhibit the ERK signaling pathway to slow myocardial fibroblast proliferation and migration [139]. Currently, there is limited research exploring the relationship between endogenous SO_2_ and fibrosis, primarily focusing on myocardial fibrosis.

Beyond myocardial fibrosis, substantial research has explored the relationship between sulfur dioxide (SO_2_) and various cardiovascular diseases. SO_2_ is endogenously produced by the catalytic action of aspartate aminotransferase 2 (AAT2) and exerts a crucial regulatory influence on the progression of cardiovascular diseases [140]. The literature indicates that downregulation of the endogenous SO_2_/AAT2 pathway may contribute to the development of myocardial hypertrophy and the senescence of cardiomyocytes [140,141]. In 2009, findings were published suggesting that endogenous SO_2_ could elevate lipid peroxide levels while diminishing glutathione (GSH), implicating its role in the pathogenesis of cardiac ischemia–reperfusion (I/R) injury in isolated rat hearts [142]. Subsequently, in 2011, studies utilizing sodium sulfite and sodium bisulfite (Na_2_SO_3_/NaHSO_3_ as SO_2_ donors) revealed that the endogenous SO_2_/glutamic oxaloacetic transaminase (GOT) pathway was implicated in the pathogenesis of isoproterenol-induced myocardial injury [143]. However, numerous unidentified targets and mechanisms still necessitate further investigation.

Expanding on this, the elucidation of the physiological roles and mechanisms of endogenous SO_2_ in fibrosis may hold significant therapeutic implications beyond myocardial fibrosis. Further exploration could shed light on its potential applications in mitigating fibrotic processes in various organs and systems and offer novel therapeutic strategies for fibrosis-related conditions. Additionally, understanding the intricate signaling pathways and molecular targets influenced by endogenous SO_2_ may unveil new therapeutic targets for intervention in fibrotic diseases, paving the way for the development of targeted therapies with improved efficacy and safety profiles.

## 7. Conclusions and Perspectives

**Figure 6:**
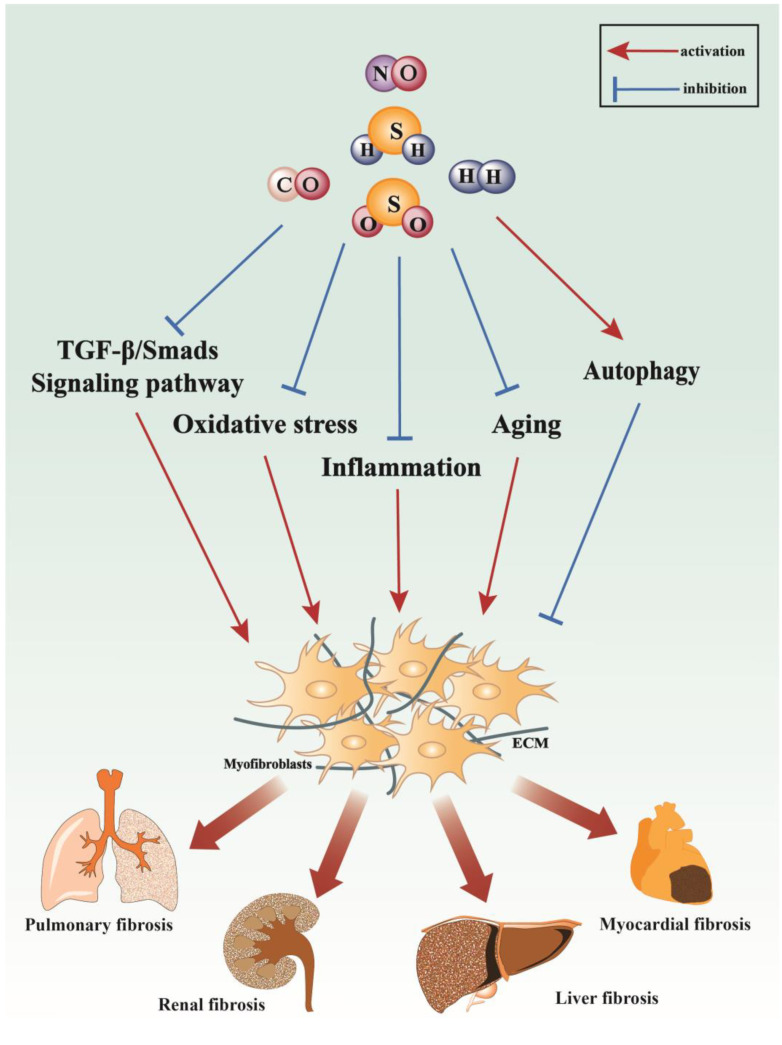
The modulation of fibrotic mechanisms involves five gasotransmitters, including NO, CO, H_2_S, H_2_, and SO_2_, which target pathways such as the TGF-β/Smads signaling pathway, oxidative stress, inflammation, aging, and autophagy to attenuate fibrotic diseases. These gasotransmitters primarily inhibit TGF-β/Smads signaling, oxidative stress, inflammation, and aging while promoting autophagy to counteract the progression of fibrosis in various organs, including the lung, kidney, heart, and liver.

Extensive research into gasotransmitters has yielded valuable insights into their involvement in the pathogenesis of fibrotic diseases. Factors such as the activation of the transforming growth factor-beta (TGF-β) signaling axis, oxidative stress, and chronic inflammation have emerged as pivotal contributors to the initiation and progression of fibrotic conditions. Among these gasotransmitters, including NO, CO, H_2_S, H_2_, and SO_2_, their diverse effects mediated through anti-inflammatory, antioxidative, anti-apoptotic mechanisms, and induction of autophagic processes have been recognized as significant factors in reducing the activation of fibroblasts and ultimately attenuating the progression of fibrosis (Figure 6). Furthermore, investigations into the molecular pathways underlying the antifibrotic effects of newer gasotransmitters such as H_2_ and SO_2_ are currently underway, offering potential therapeutic avenues. Notably, inhaled NO therapy has demonstrated a broad spectrum of clinical applications in treating various pulmonary, cardiac, neural, and infection-related conditions [112]. Similarly, clinical trials exploring the use of inhaled CO for IPF patients have shown promising results [113]. While numerous animal models and experimental studies have provided compelling evidence of the beneficial effects of gasotransmitters on fibrotic diseases, rigorous clinical trials are indispensable to validate these findings and ascertain their potential translation into clinical practice. Consequently, the initiation of well-designed clinical trials is paramount to confirming and further elucidating the therapeutic potential of gasotransmitters in the treatment and management of fibrotic conditions. These endeavors underscore the ongoing commitment to fully harnessing the therapeutic potential of gasotransmitters in combating fibrotic diseases.

To achieve a comprehensive understanding of the intricate mechanisms underlying the effects of gasotransmitters in fibrotic diseases, additional experiments and research endeavors are imperative. Unraveling these mechanisms will be pivotal in shaping more targeted and efficacious therapeutic interventions for fibrotic conditions. Scientists and researchers are actively engaged in elucidating the complex signaling pathways and molecular interactions that underlie the modulation of fibrosis by gasotransmitters. Concurrently, the exploration of novel exogenous gasotransmitter donors represents a critical area of investigation (Table 1). The development of these donors offers a potent tool for manipulating gasotransmitter levels within specific tissues or organs, enabling precise control over their therapeutic actions. This approach holds significant promise for overcoming challenges associated with endogenous gasotransmitter production, thereby ensuring optimal therapeutic outcomes. Moreover, given the multifaceted nature of fibrotic diseases, a combinatorial therapy approach involving exogenous gasotransmitter donors may prove to be a viable strategy. By concurrently targeting multiple pathways, this approach has the potential to enhance therapeutic efficacy and potentially attenuate the progression of fibrosis. Rigorous clinical trials aimed at assessing the safety and effectiveness of combined treatments are warranted to ascertain the viability and impact of such strategies.

Overall, the emerging recognition of both endogenous and exogenous gasotransmitters as promising therapeutic targets in fibrogenesis underscores the need for further exploration. Through ongoing research efforts, novel insights into the precise mechanisms of action can be uncovered, paving the way for innovative strategies to harness the therapeutic potential of gasotransmitters in clinical settings. As researchers continue to refine and advance these approaches, gasotransmitters stand poised to offer valuable contributions to the arsenal against fibrotic diseases.

## Figures and Tables

**Table 1:** The conclusion of five gasotransmitters administration, corresponding diseases, and precise mechanism in fibrosis.

Gasotransmitters	Administration	Diseases	Mechanisms	Quotation
NO	SNAP	Liver fibrosis	SNAP eliminates ROS production, inhibits the production of ECM.	[15]
		Liver fibrosis	SNAP donors promote HSC apoptosis and maintains sinus homeostasis by producing superoxide and hydroxyl radical intermediates.	[16]
		Renal fibrosis	SNAP inhibits the proliferation of MCs, and down-regulates the expression of ECM.	[36]
		Renal fibrosis	SNAP reduces fibrosis formation by inhibiting ECM synthesis.	[7,38,39]
		Renal fibrosis	SNAP alleviates fibrosis by regulating the expression of several ECM-degrading proteases and intrinsic inhibitors in renal MCs, including MMP-9, MMP-13, plasminogen activator inhibitor-1 (PAI-1), and TIMP-1.	[40,41]
		Renal fibrosis	SNAP exerts antifibrotic effects by amplifying TIMP-1 expression in renal MCs in a TGF-β-dependent manner.	[42]
	SNP	Liver fibrosis	SNP inhibits CCl4 induced liver fibrosis via enhancing MSC.	[17]
CO	CO gas	Idiopathic pulmonary fibrosis	CO gas upregulates the expression of p21 Cip1 to inhibit the proliferation of fibroblasts.	[57]
		Renal fibrosis	250 ppm CO activates MKK3 signaling pathway to inhibit the development of renal fibrosis and protect the kidney.	[62]
		Renal fibrosis	CO suppresses the expression of TGF-β1/Smads and ERK-MAPK pathways to inhibit progression of fibroinflammatory process of CAN.	[63]
		Myocardial fibrosis	CO inhibits TGF-β signaling and stimulating autophagy.	[64]
	CORM-A1	Idiopathic pulmonary fibrosis	CORM-A1 reduces the expression of profibrogenic cytokines COX-2, TNF-α, and α-SMA and serum hydroxyproline to alleviate paraquat-induced pulmonary fibrosis.	[58]
	SMA/CORM2	Liver fibrosis	SMA/CORM2 inhibits HIF-1α-mediated inflammatory cascade.	[66]
	CORM2	Liver fibrosis	CORM2 up-regulates TTP to reduce the level of PAI-1 in NAFLD.	[70]
	CO-HbV	Idiopathic pulmonary fibrosis	CO-HbV attenuates IPF through suppressing NOX4 activity to reduce ROS, decreasing TGF-β1 and inflammatory cytokines containing TNF-α, IL-1β, IL-6 in the lung.	[46]
H_2_S	NaHS	Renal fibrosis	NaHS suppresses the levels of TGF-β1/Smads and NF-κB signaling pathway.	[83]
		Renal fibrosis	NaHS inhibits the differentiation of renal fibroblasts into myoblasts by inhibiting TGF-β1/Smads and MAPK signaling pathways.	[82]
		Renal fibrosis	NaHS ameliorates renal fibrosis by inhibition of M1 and M2 macrophage infiltration and NLRP3 activation and subsequently NF-κB and IL-4/STAT6 signaling.	[84]
		Renal fibrosis	NaHS up-regulates the expression of CX40, CX43 and CX45 and regulating MMPs/TIMPs parameters.	[86]
		Renal fibrosis	NaHS improves renal tissue fibrosis by inhibiting autophagy, upregulating SOD and downregulating AKT, TGF-β1 and NF-κB in diabetic rats.	[87]
		Renal fibrosis	NaHS inhibits fibrosis by inhibiting the ERK1/2 pathway and TGF-β1 signaling.	[88]
		Myocardial fibrosis	NaHS modulates MMPs/TIMPs and TGF-β1 expression to attenuate cardiac fibrosis.	[94]
		Myocardial fibrosis	NaHS ameliorates myocardial fibrosis by decreasing myocardial autophagy and activating the PI3K/AKT1 pathway.	[96]
		Myocardial fibrosis	NaHS attenuates myocardial fibrosis in diabetic rats by down-regulating JAK-STAT signaling pathway, thereby inhibiting oxidative stress and ER stress, inflammatory response and apoptosis.	[98]
		Myocardial fibrosis	NaHS ameliorates thyroxin-induced myocardial fibrosis by up-regulating the expression of PI3K/AKT signaling pathway and down-regulating the expression of miR-21, miR-34a and miR-214.	[99]
		Myocardial fibrosis	NaHS inhibits MMPs/TIMPs expression, inflammatory response and alleviates myocardial fibrosis by regulating PKC-ERK1/2MAPK signaling pathway.	[100]
		Myocardial fibrosis	NaHS inhibits myocardial fibrosis by down-regulating the typical Wnt and TGF-β1/Smad3 pathways, reducing myocardial collagen deposition.	[101]
		Myocardial fibrosis	NaHS inhibits doxorubicin-induced myocardial fibrosis by inhibition of overactivation of the ER and that of autophagy via upregulation of the PI3K/AKT/mTOR pathway.	[102]
		Myocardial fibrosis	NaHS delays myocardial fibrosis by increasing the expression of miR-133a, and CTGF.	[103]
		Myocardial fibrosis	NaHS inhibits necroptosis and alleviates hypoxia-induced cardiac fibroblast proliferation via SIRT3.	[104]
		Liver fibrosis	NaHS inhibits the proliferation of activated hsc, induce cell cycle arrest and apoptosis, and weaken CCL4-induced liver fibrosis and ECM expression.	[9]
		Liver fibrosis	NaHS inhibits the formation of liver fibrosis by down-regulating AGTR1 expression.	[107]
		Idiopathic pulmonary fibrosis	NaHS attenuates pulmonary fibrosis possibly through reducing oxidative stress.	[110]
		Idiopathic pulmonary fibrosis	NaHS induces the nuclear accumulation of Nrf2 in lung tissue of smoking rats, thereby up-regulating the expression of antioxidant genes HO-1 and Trx-1 and inhibiting pulmonary fibrosis.	[113]
	H_2_S gas	Idiopathic pulmonary fibrosis	H_2_S inhibits the progression of EMT in alveolar epithelial cells and alleviates pulmonary fibrosis by reducing the phosphorylation of Smad2/3.	[111]
		Idiopathic pulmonary fibrosis	H_2_S inhibits fibrosis by inhibiting NF-κB p65 expression and regulating Th1/Th2 balance.	[112]
	NaHS, GYY4137	Liver fibrosis	The exogenous and endogenous H_2_S increases the proliferation and activation of HSCs, and suppressed H_2_S can decrease the proliferation and fibrotic marks of HSCs.	[108]
	Hydrogen gas	Idiopathic pulmonary fibrosis	Hydrogen gas inhibits pulmonary fibrosis by reducing the production of ROS and TGF-β.	[119]
Hydrogen		Pulmonary fibrosis	Hydrogen gas restrains the expression of IL-6, IL-4 and IL-13, and suppressing M2-biased macrophage polarization to reduce alveolar fibrosis.	[120]
		Myocardial fibrosis	Hydrogen gas ameliorates myocardial fibrosis mainly by inhibiting NLRP3-mediated pyroptosis.	[125]
		Peritoneal fibrosis	Hydrogen gas exerts anti-peritoneal fibrosis effect through inhibiting ROS/PTEN/AKT/mTOR pathway.	[133]
		Myocardial fibrosis	Hydrogen gas decreases the level of ROS and the phosphorylation of p38 MAPK, Smad2/3, the expression of TGF-β1 and CTGF to reduce myocardial fibrosis.	[123]
	Hydrogen Water	Myocardial fibrosis	Hydrogen Water prevents LPS-induced EMT and pulmonary fibrosis by reducing α-SMA production and inhibiting oxidative stress.	[118]
		Liver fibrosis	Molecular hydrogen inhibits liver fibrosis by interacting with HO1/IL-10, inhibiting the expression of phosphorylated STAT3 and activation of downstream pMAPK signals.	[128]
		Renal fibrosis	Hydrogen Water reduces the fibrosis and infiltration of macrophage in UUO kidneys by decreasing MDA level and increasing SOD activity.	[130]
		Renal fibrosis	Hydrogen Water reduces renal fibrosis by increasing the expression of Sirt1, a downstream molecule of TGF-β1.	[10]
		Peritoneal fibrosis	Hydrogen Water through inhibiting the expression of TGF-β1 and PAI-1, increasing the expression of t-PA, and reducing oxidative stress and inflammation.	[131]
SO_2_	Na_2_SO_3_/NaHSO_3_	Myocardial fibrosis	SO_2_ attenuates myocardial fibrosis in diabetic rats by down-regulating Hippo-MST pathway to reduce apoptosis and ER stress.	[135]

## Abbreviations

Acute respiratory distress syndrome (ARDS), acute respiratory distress (ARD), alveolar concentration of nitric oxide (CaNO), carbon monoxide (CO), chronic allograft nephropathy (CAN), chronic kidney disease (CKD), CO-bound hemoglobin vesicles (CO-HbV), cystathionine-β-synthase (CBS), cystathionine-γ-lyase (CSE), D-amino acid oxidase (DAO), endothelial NOS (eNOS; NOS3), end-stage renal disease (ESRD), epithelial–mesenchymal transition (EMT), extracellular matrix (ECM), fibronectin (FN), heme oxygenase (HO), hepatic stellate cells (HSCs), high-fat methionine- and choline-deficient (HF-MCD), human immunodeficiency virus (HIV), human kidney proximal tubular epithelial cells (HK-2 cells), hydrogen (H_2_), hydrogen sulfide (H_2_S), hydrogen-containing saline (HCS), hydrogen-rich saline (HRS), hypoxia-inducible factor 1-alpha (HIF-1α), idiopathic pulmonary fibrosis (IPF), inducible NOS (iNOS; NOS2), inhaled nitric oxide (iNO), inhibitor of DNA binding 1 (Id1), interleukin10 (IL-10), Lipopolysaccharide (LPS), 3-mercaptopyruvate sulphurtransferase (3-MST), matrix metallopeptidase 9 (MMP-9), mesangial cells (MCs), metabolic-associated fatty liver disease (MAFLD), neuronal NOS (nNOS; NOS1), nitric oxide (NO), nitric oxide synthases (NOSs), non-alcoholic fatty liver disease (NAFLD), Nuclear factor erythroid 2-related factor 2 (Nrf2), peritoneal dialysis (PD), plasminogen activator inhibitor-1 (PAI-1), plasminogen activator inhibitor-1 (PAI-1), postsurgical peritoneal adhesions (PPAs), recombinant Angiotensin II Receptor 1 (AGTR1), S-allyl-cysteine (SAC), small proline-rich protein-1a expression (Sprr1a), S-nitroso-N-acetyl-D,L-penicillamine (SNAP), sodium hydrosulfide (NaHS), sodium sulfide (Na_2_S), stress granules (SGs), sulfur dioxide (SO_2_), ten-eleven translocation (TET), tristetraprolin (TTP), unilateral ureteral obstruction (UUO).
